# Determination of Risk Factors for Postoperative Acute Kidney Injury in Patients With Gynecologic Malignancies

**DOI:** 10.7759/cureus.41836

**Published:** 2023-07-13

**Authors:** Melek Doganci, Pınar Zeyneloğlu, Zeynep Kayhan, Ali Ayhan

**Affiliations:** 1 Department of Critical Care, Ataturk Chest Diseases and Thoracic Surgery Training and Research Hospital, Ankara, TUR; 2 Department of Anesthesiology and Intensive Care Unit, Başkent University Faculty of Medicine, Ankara, TUR; 3 Department of Anesthesiology and Reanimation, Başkent University Faculty of Medicine, Ankara, TUR; 4 Department of Obstetrics and Gynecology, Başkent University Faculty of Medicine, Ankara, TUR

**Keywords:** acute kidney injury, prolonged operation times, gynecological malignancy, gynecologic oncology surgery, gynecological cancer

## Abstract

Background

Postoperative acute kidney injury (AKI) is an important cause of mortality and morbidity among surgical patients. There is little information on the occurrence of AKI after operations for gynecologic malignancies. This study aimed to determine the incidence of AKI in patients who underwent surgery for gynecological malignancies and determine the risk factors in those who developed postoperative AKI.

Methodology

A total of 1,000 patients were enrolled retrospectively from January 2007 to March 2013. AKI was defined according to the Kidney Disease Improving Global Outcomes 2012 Clinical Practice Guideline for Acute Kidney Injury. Perioperative variables of patients were collected from medical charts.

Results

The incidence of postoperative AKI was 8.8%, with stage 1 occurring in 5.9%, stage 2 in 2.4%, and stage 3 in 0.5% of the patients. Patients who had AKI were significantly older, had higher body mass index (BMI) higher preoperative C-reactive protein (CRP) levels, and more frequently had a history of distant organ metastasis when compared with those who did not have AKI. When compared with patients who did not develop AKI postoperatively, longer operation times and intraoperative usage of higher amounts of erythrocyte suspension and fresh frozen plasma were seen in those who developed AKI.

Conclusions

Patients who had AKI were older, had higher BMI with higher preoperative CRP levels, more frequent distant organ metastasis, longer operation times, and higher amounts of blood transfused intraoperatively. Defining preoperative, intraoperative, and postoperative risk factors for postoperative AKI and taking necessary precautions are important for the early detection and intervention of AKI.

## Introduction

Currently, cancer is the second most common cause of death. Gynecological cancers lead to significant morbidity and mortality in women after breast cancer. Gynecological cancers are very common in our country with multidimensional negative effects on the health of affected women. Although the survival rate is generally good in women who undergo gynecological cancer surgeries, postoperative complications can occur. One of the complications is acute kidney injury (AKI) which affects the quality of life, leading to morbidity and mortality. Despite the renal treatment, the mortality associated with AKI is high because patients are generally elderly and weak due to comorbidities [1].

Postoperative AKI is known to be associated with individual factors such as age, genetic predisposition, a history of kidney failure, type 1 diabetes mellitus (DM), cirrhosis, mechanical ventilation-induced lung injury, rhabdomyolysis, and tumor lysis syndrome. Moreover, anesthesia and surgery also significantly affect the development of AKI.

Although many studies have investigated AKI after cardiac surgery and kidney transplantation, there is limited information on postoperative AKI in patients undergoing surgeries for gynecological cancer.

Serious risk factors for postoperative AKI in gynecological cancer surgeries include intraoperative hypotension, hypovolemia, massive blood transfusion requirement, ureter injury, increased intra-abdominal pressure, prolonged surgery duration, and surgical method (debulking, cytoreductive surgery, and laparoscopic surgery) [2,3]. However, studies investigating these risk factors are limited and conducted on a small number of patients [4].

Therefore, this study aims to retrospectively evaluate postoperative kidney function as well as preoperative, intraoperative, and postoperative risk factors for AKI in 1,000 patients who underwent surgery for gynecological cancer.

## Materials and methods

Medical and anesthesia recordings of patients undergoing elective gynecological cancer surgery at Başkent University, Ankara Hospital between January 2007 and March 2013 were retrospectively analyzed after obtaining approval from Başkent University Medical and Health Sciences Research Board (approval number: KA12/189). The study included 1,000 patients who were examined in the services and/or intensive care unit (ICU), who were over the age of 18 years, and those without kidney damage or disease. As premedication, 1-2 mg midazolam was administered preoperatively.

According to the drugs chosen by the anesthesiologist, maintenance anesthesia was provided with intravenous (IV) induction of general anesthesia, followed by an inhalation agent. After performing proper sterile cleaning and draping in spinal anesthesia, a regional block with a local anesthetic was applied at the L3-4/L4-5 interval. For epidural anesthesia, after the insertion of the epidural catheter, a regional block was achieved by a local anesthetic agent. The surgical operation started after the completion of necessary invasive monitoring procedures in patients with American Society of Anesthesiologists (ASA) Grade 3 and above and who were planned for major surgery. In this study, hemodynamic and respiratory parameters (such as systolic and diastolic blood pressure, heart rate and oxygen saturation, body temperature, and end-tidal CO_2_ monitoring), urine output rate per hour, and blood loss were recorded. If blood gas monitoring was necessary, on average, blood gas is taken from patients twice or thrice. During surgery, patients were hydrated with appropriate fluid, and in case of need for erythrocyte suspension (ES), fresh frozen plasma (FFP) transfusions were performed.

In the preoperative period, patients’ age, height, body mass index (BMI), ASA score, systemic comorbidities (such as cardiovascular system disease, respiratory system disease, DM, hematological diseases, and central nervous system diseases), the drugs used (such as antihypertensives, oral antidiabetics, insulin preparations, antibiotics, diuretics, non-steroidal anti-inflammatory drugs, and contrast agents), preoperative lab results (such as hematocrit, platelet, blood urea nitrogen (BUN), serum creatinine, serum sodium, serum potassium, serum calcium, total bilirubin, aspartate aminotransferase (AST), alanine aminotransferase (ALT), prothrombin time (PT), international normalization ratio (INR), albumin, protein and C-reactive protein (CRP)), presence of metastasis, chemotherapy/radiotherapy anamnesis, vital signs (body temperature, heart rate, and blood pressure) and recurrent surgeries were recorded.

In the intraoperative period, anesthesia method (such as general, regional anesthesia, or general plus regional anesthesia), anesthetic agents used, type of surgery, vital signs, fluids administered (such as crystalloids and colloids), blood transfusion (such as albumin as colloid, gelofusine, and fresh frozen plasma), critical incidents (hypotension (blood pressure <90/60 mmHg), hypertension (blood pressure >140/90 mmHg), bradycardia (heart rate <60 beats/minute), tachycardia (heart rate >100 beats/minute), bleeding, hypoxemia (saturation <92%), hypothermia (<36°C), hypocapnia (PaCO_2_ <35 mmHg) and hypercapnia (PaCO_2_ >45 mmHg), blood loss (calculated by the aspirator and number of compress and sponges), complications (such as ureteral damage, suspected pulmonary thromboembolism, vascular trauma, bowel perforation, and organ injury), inotropic agents requirement, and surgery duration were documented.

In the postoperative period, abnormal vital signs in the first 24 hours, crystalloids and colloids administered in the first 48 hours, urinary/non-urinary infections, complications (such as respiratory, cardiac, neurologic, and hematological), urinary output in the first 24 hours, nephrotoxic drugs used, laboratory results in the first seven days (such as BUN, serum creatinine, serum sodium, serum potassium, hematocrit, platelet, AST, ALT, bilirubin, albumin, PT, INR, and CRP), length of hospital stay, ICU requirement, length of ICU stay, need for invasive mechanical ventilation, mortality, and renal replacement therapy indication were recorded.

SPSS version 20 is used for the statistical analyses. To diagnose AKI and identify the degree of damage, the Kidney Disease Improving Global Outcomes (KDIGO) 2012 guideline was used. Unless specified otherwise, continuous data were described as mean ± SD for normal distributions and median (interquartile range) for skewed distributions. Categorical data were described as the number of cases (%). For the comparison between patients with and without AKI, the chi-square test was used for variables such as systemic disease and drugs used. Moreover, the Mann-Whitney test was used to compare numerical values. Parameters that differed statistically between the two groups (p < 0.05) and differed between the two groups according to our clinical observation were included in the logistic regression analysis.

## Results

Demographic features of 1,000 patients undergoing surgery for gynecological cancer were examined. The average age was 55.4 ± 12.4 years, the average weight was 75.1 ± 16.2 kg, the average height was 162. 1 ± 5.5 cm, and the average BMI was 28.7 ± 6.4 kg/m^2^. Patients were categorized into two groups according to their ASA scores, i.e., ASA Grades 1-2 (911 patients) and ASA Grades 3-4 (89 patients) for statistical comparison. Overall, 40,7% of participants had systemic comorbidities, with hypertension and DM being the most common. A history of preoperative regular drug use was found in 53.4% of the patients, most commonly antihypertensives (37.5%). Further, 15.2 of the patients were exposed to contrast agents preoperatively. Overall, 30.3% of patients underwent recurrent surgery, 18.8% had preoperative metastasis, 12.9% underwent preoperative chemotherapy, and 5% underwent preoperative radiotherapy.

General anesthesia was performed in 97% of the patients, spinal anesthesia in 1.2%, combined (spinal + epidural) anesthesia in 0.9%, general and regional anesthesia in 0.8%, and epidural anesthesia in 0.1% of patients. In addition, among the anesthetics used in patients, IV agents included 60.9% thiopental, 36.5% propofol, 1.8% etomidate, and 0.8% ketamine, and inhalation agents included 42.6% sevoflurane, 32.3% isoflurane, and 25.1% desflurane.

**Figure 1:**
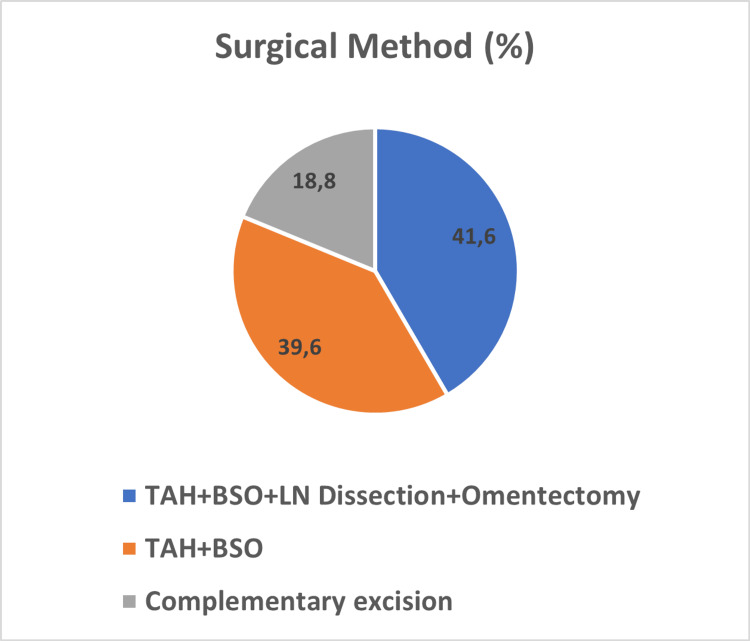
Surgical method (%). TAH = total abdominal hysterectomy; BSO = bilateral salpingo-oophorectomy; LN = lymph node

Total abdominal hysterectomy (TAH) and bilateral salpingo-oophorectomy (BSO) were performed in 39.6% of the patients; wide surgical excisions such as TAH, BSO, lymph node dissection, omentectomy, and appendectomy were performed in 41.6%; and complementary excision was performed in 18.8% of patients (Figure 1).

Postoperatively, 11% of the 1,000 patients needed ICU admission, and 2.5% of patients needed invasive mechanical ventilation. The hospital stay of the patients was a minimum of two days and a maximum of 60 days, with a mean duration of 9.8 ± 7.3 days. Furthermore, ICU stay was a minimum of one day and a maximum of 38 days, with a mean duration of 3.3 ± 4.6 days. AKI was in 88 of 1,000 patients (88%) (59 patients in stage 1, 24 patients in stage 2, and 5 patients in stage 3). Only one (0.1%) patient needed renal replacement therapy. The postoperative mortality in 28 days was 0.5%.

On comparing the demographic factors of patients with and without AKI, patients with AKI were older (p = 0.046), shorter (p = 0.044), and had higher BMI (p = 0.031) than patients without AKI. However, regarding weight, significant differences were not found (p = 0.145). Systemic comorbidities and preoperative drug use were similar between patients with and without AKI (p > 0.05) (Table 1).

**Table 1:** Demographic characteristics of patients with and without acute kidney injury (mean ± standard deviation). Continuous variables are expressed as either the mean ± standard deviation (SD) or median (interquartile range) and categorical variables are expressed as either frequency (percentage). Continuous variables were compared with Student’s t-test or Mann-Whitney U test, and categorical variables were compared using Pearson’s chi-square test or Fisher’s exact test. Statistically significant p-values are in bold. BMI = body mass index; CAD = coronary artery disease; DM = diabetes mellitus; HT = hypertension; COPD = chronic obstructive pulmonary disease; CNS: central nervous system; ASA: American Society of Anesthesiologists; OAD = oral anti-diabetics; NSAIDs = non-steroidal anti-inflammatory drugs

	AKI (+) (n = 88)	AKI (-) (n = 912)	P-value
Age (year)	57.9 ± 12.8	55.1 ± 12.3	0.046
Weight (kg)	77.5 ± 16.8	74.9 ± 16.1	0.145
Height (cm)	161.0 ± 6.1	162.2 ± 5.4	0.044
BMI (kg/m^2^)	30.1 ± 7.1	28.6 ± 6.4	0.031
Comorbidities	54 (61.4)	539 (59.1)	0.734
CAD	9 (10.2)	72 (7.9)	0.415
DM	15 (17)	169 (18.5)	0.885
HT	40 (45.5)	360 (39.5)	0.305
COPD	3 (3.4)	35 (3.8)	1.00
Anemia	2 (2.3)	54 (5.9)	0.222
CNS disease	5 (5.7)	45 (4.9)	0.796
ASA score			
ASA 1-2	78 (88.6)	833 (91.3)	0.430
ASA 3-4	10 (11.4)	79 (8.7)	0.430
Preoperative drug use	50 (56.8)	484 (53.1)	0.576
Antihypertensive	41 (46.6)	334 (36.6)	0.083
OAD	11 (12.5)	116 (12.7)	1.00
Insulin	1 (1.1)	26 (2.9)	0.504
Contrast agent	19 (21.6)	133 (14.6)	0.087
Antibiotics	6 (6.8)	42 (4.6)	0.304
Diuretics	4 (4.5)	35 (3.8)	0.770
NSAIDs	6 (6.8)	49 (5.4)	0.621

According to preoperative laboratory results, patients with AKI had higher CRP values (p = 0.037). However, there are no statistically significant differences between other laboratory results and AKI progression (p > 0.05) (Table 2).

**Table 2:** Comparison of preoperative laboratory data of patients with and without acute kidney injury (mean ± standard deviation). Continuous variables are expressed as either the mean ± standard deviation (SD). Continuous variables were compared with Student’s t-test or Mann-Whitney U test. Statistically significant p-values are in bold. BUN = blood urea nitrogen; Na^+^ = sodium; K^+^ = potassium; Ca^+2^ = calcium; CRP = C-reactive protein; ALT = alanine aminotransferase; AST = aspartate aminotransferase; PT = prothrombin time; INR = international normalized ratio

	AKI (+) (n = 88)	AKI (-) (n = 912)	P-value
Hematocrit (%)	36.6 ± 4.2	37.0 ± 4.5	0.427
Platelets (/mm^3^)	320,648.9 ± 13,111.6	315,181.3 ± 110,246.7	0.663
BUN (mg/dL)	15.9 ± 9.0	15.2 ± 7.0	0.367
Creatinine (mg/dL)	0.7 ± 0.3	0.7 ± 0.2	0.134
Na^+^ (mmol/L)	139.0 ± 3.1	139.0 ± 3.1	0.849
K^+^ (mmol/L)	4.0 ± 0.6	4.1 ± 0.5	0.879
Ca^+2^ (mg/dL)	8.8 ± 0.7	9.2 ± 0.7	0.468
CRP (mg/L)	157.8 ± 121.5	76.6 ± 81.1	0.037
ALT (U/L)	22.0 ± 36.2	21.1 ± 22.3	0.733
AST (U/L)	24.5 ± 20.2	23.5 ± 24.4	0.722
PT (seconds)	13.4 ± 1.7	13.2 ± 1.6	0.399
INR	1.0 ± 0.2	1.0 ± 0.2	0.502
Albumin (g/dL)	3.3 ± 0.5	3.3 ± 0.6	0.570

While AKI is diagnosed more often in patients with distant metastasis in the preoperative period (p = 0.022), no significant differences were noted in patients who had a preoperative history of chemotherapy, radiotherapy, and infection, as well as a history of repeated recurrent surgery (p > 0.05). On comparing participants’ preoperative vital signs, patients with higher heart rates had a high incidence of AKI (p = 0.026). In contrast, there was no significant difference between patients with and without AKI regarding blood pressure and body temperature measurements (p > 0.05) (Table 3).

**Table 3:** Preoperative characteristics of patients with and without acute kidney injury (number (%)). Continuous variables are expressed as either the mean ± standard deviation (SD) or median (interquartile range) and categorical variables are expressed as frequency (percentage). Continuous variables were compared with Student’s t-test or Mann-Whitney U test, and categorical variables were compared using Pearson’s chi-square test or Fisher’s exact test. Statistically significant p-values are in bold.

	AKI (+) (n = 88)	AKI (-) (n = 912)	P-value
Presence of preoperative metastases	25 (28.4)	163 (17.9)	0.022
Preoperative chemotherapy	17 (19.3)	112 (12.3)	0.067
Preoperative radiotherapy	3 (3.4)	47 (5.2)	0.614
Presence of preoperative infection	7 (8)	34 (3.7)	0.082
Repetitive surgery	34 (38.6)	269 (29.5)	0.089
Body temperature (°C)	36.5 ± 0.5	36.4 ± 0.4	0.218
Systolic blood pressure (mmHg)	121.5 ± 15.4	119.6 ± 11.7	0.169
Diastolic blood pressure (mmHg)	74.9 ± 8.3	74.3 ± 8.3	0.539
Mean arterial pressure (mmHg)	90.0 ± 9.8	89.0 ± 8.4	0.315
Pulse rate (beats/minute)	84.0 ± 12.0	80.8 ± 10.6	0.026

On comparing the different anesthesia methods, no significant difference was noted in patients with or without AKI (p > 0.05) (Table 4).

**Table 4:** Anesthesia methods in patients with and without acute kidney injury (number (%)). Categorical variables are expressed as either frequency (percentage). Categorical variables were compared using Pearson’s chi-square test or Fisher’s exact test. Statistically significant p-values are in bold.

Anesthesia methods	AKI (+) (n = 88)	AKI (-) (n = 912)	P-value
General anesthesia	86 (97.7)	884 (96.9)	1.000
Spinal anesthesia	0 (0)	12 (1.3)	0.614
Combined (spinal + epidural anesthesia)	1 (1.1)	8 (0.9)	0.565
Epidural anesthesia	0 (0)	1 (0.1)	1.000
General + regional anesthesia	1 (1.1)	7 (0.8)	0.523

**Figure 2:**
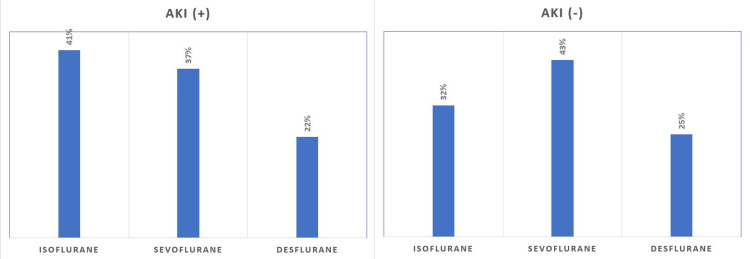
Anesthetic inhalation agents used in patients with and without acute kidney injury.

**Figure 3:**
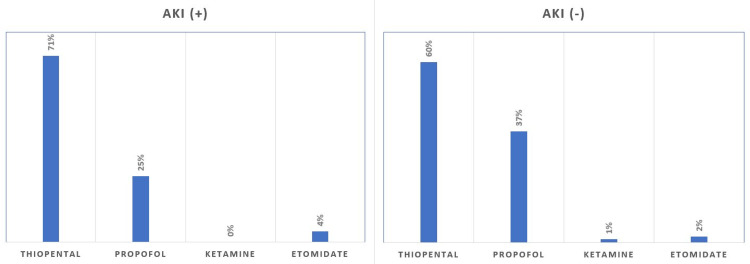
Intravenous anesthetic agents used in patients with and without acute kidney injury.

In addition, there was no significant difference between inhalation and intravenous agents used intraoperatively (p > 0.05). Of the 88 patients with AKI, isoflurane was used in 41%, sevoflurane in 37%, and desflurane in 22% of patients, and intravenous anesthetic agents included 71% thiopental, 25% propofol, and 4% etomidate (Figures 2, 3).

The study patients were categorized into the following two groups according to surgical methods: those who did not undergo extensive surgery (TAH + BSO operated) and those who underwent extensive surgery (TAH + BSO + LN dissection + wide surgical excision such as omentectomy and those who underwent complementary excision). There was a statistically significant difference between patients with and without extensive surgery in terms of AKI progression (p < 0.001). In addition, surgery duration was longer in patients who had AKI (p < 0.001). The total amount of crystalloid used in the intraoperative period was similar in patients with and without AKI (p = 0.240). On the other hand, the total amount of colloid, ES, and FFP use was statistically significantly different between the groups (p = 0.043, p = 0.001, and p = 0.001, respectively). Furthermore, patients with AKI experienced more blood loss than those without AKI (p = 0.011). When the groups were examined in terms of intraoperative problems, among the intraoperative surgical complications, vascular trauma and/or leakage from large dissection surfaces were found to be higher in patients with AKI than in those without AKI (p < 0.05). The two groups were similar in terms of other intraoperative problems (p > 0.05) (Table 5).

**Table 5:** Examination of the intraoperative period in patients with and without acute kidney injury (mean ± standard deviation or number (%)). Continuous variables are expressed as either the mean ± standard deviation (SD) or median (interquartile range) and categorical variables are expressed as frequency (percentage). Continuous variables were compared with Student’s t-test or Mann-Whitney U test, and categorical variables were compared using Pearson’s chi-square test or Fisher’s exact test. Statistically significant p-values are in bold. ES = erythrocyte suspension; FFP = fresh frozen plasma; BP = blood pressure; PTE: pulmonary thromboembolism

	AKI (+) (n = 88)	AKI (-) (n = 912)	P-value
Extensive surgery	70 (79.5)	534 (58.6)	0.001
No extensive surgery	18 (20.5)	378 (41.4)	
Surgery time (minutes)	149.1 ± 62.5	123.8 ± 56.6	0.001
Total crystalloid used (mL/kg)	38.4 ± 19.9	35.9 ± 18.8	0.240
Total colloid used (mL/kg)	4.0 ± 5.0	2.9 ± 4.3	0.043
Total ES used (mL)	279.5 ± 616.7	128.3 ± 296.1	0.001
Total FFP used (mL)	165.9 ± 284.8	94.1 ± 185.9	0.001
Need for intraoperative inotropes	9 (10.2)	66 (7.2)	0.291
Volume of intraoperative blood loss	517.9 ± 560.9	391.5 ± 420.8	0.011
İntraoperative urine volume (mL)	275.1 ± 238.1	245.6 ± 237.3	0.313
Hypotension (BP <90/60 mmHg)	25 (28.4)	207 (22.7)	0.235
Hypertension (BP >140/90 mmHg)	60 (68.2)	550 (60.3)	0.170
Hypoxemia (saturation <92%)	5 (5.7)	70 (7.7)	0.671
Hypothermia (<36°C)	7 (8)	84 (9.2)	0.847
Hypercapnia (PaCO_2_ >45 mmHg)	4 (4.6)	38 (4.2)	0.782
Hypocapnia (PaCO_2_ <35 mmHg)	9 (10.3)	139 (15.4)	0.270
Ureteral damage	3 (3.4)	14 (1.5)	0.183
Suspected PTE	0 (0)	1 (0.1)	1.000
Vascular trauma/bleeding	4 (4.5)	12 (1.3)	0.045
Organ injury	1 (1.1)	14 (1.5)	1.000
Bowel perforation	8 (9.1)	51 (5.6)	0.230

Hypertension (p = 0.022) and tachycardia (p = 0.004) were more common in patients with AKI that those without AKI in the first 24 hours of the postoperative period. When the groups were compared for the use of crystalloid and colloid in the postoperative first and second days, the colloid amount used in the postoperative 48 hours was statistically significantly higher in patients with AKI (p = 0.001 and p = 0.005, respectively). Among postsurgical infections, non-urinary infections (p = 0.037), and among postoperative complications, respiratory complications (p = 0.015) were statistically significantly higher in patients with AKI, whereas cardiac, neurological, and hematological complications were similar in both groups ( p > 0.05). There was a significant decrease in urine output in the first 24 hours postoperatively in patients with AKI (p = 0.002). The need for ICU admission was more frequent (p = 0.032), the length of stay in the ICU was longer (p < 0.001), and the need for invasive mechanical ventilation was higher in patients with AKI than in those without AKI (p = 0.004). In patients with AKI, hospital stay was longer (p < 0.001). There was no difference between the two groups in terms of postoperative five-day renal replacement therapy need and postoperative 28-day mortality (Table 6).

**Table 6:** Comparison of postoperative period in patients with and without acute kidney injury (number (%) or mean ± standard deviation). Continuous variables are expressed as either the mean ± standard deviation (SD) or median (interquartile range) and categorical variables are expressed as frequency (percentage). Continuous variables were compared with Student’s t-test or Mann-Whitney U test, and categorical variables were compared using Pearson’s chi-square test or Fisher’s exact test. Statistically significant p-values are in bold. BP = blood pressure; MV = mechanical ventilation; RRT = renal replacement therapy

	AKI (+) (n = 88)	AKI (-) (n = 912)	P-value
Vital signs in the first 24 hours			
Hypotension (BP <90/60 mmHg)	4 (4.5)	41 (4.5)	1.000
Hypertension (BP >140/90 mmHg)	45 (51.1)	350 (38.4)	0.022
Tachycardia (heart rate >100beats/minute)	49 (55.7)	360 (39.5)	0.004
Bradycardia (heart rate <60 mmHg)	4 (4.5)	81 (8.9)	0.227
Hypothermia (<36°C)	13 (14.8)	122 (13.4)	0.743
Hyperthermia (>38°C)	19 (21.6)	161 (17.7)	0.383
Postoperative fluid (mL)			
Crystalloid (first day)	3,351.0 ± 1,138.9	3,191.5 ± 907.7	0.125
Crystalloid (second day)	3,560.2 ± 792.8	3,392.4 ± 869.3	0.082
Colloid (first day)	191.9 ± 411.4	98.0 ± 229.4	0.001
Colloid (second day)	91.6 ± 246.7	42.8 ± 142.8	0.005
Urinary infection	11 (12.5)	62 (6.8)	0.081
Non-urinary infection	17 (19.3)	102 (11.2)	0.037
Presence of complications			
Respiratory	7 (8)	24 (2.6)	0.015
Cardiac	5 (5.7)	27 (3)	0.193
Neurological	2 (2.3)	13 (1.4)	0.386
Hematological	13 (14.8)	76 (8.3)	0.050
Nephrotoxic drug use	71 (80.7)	664 (72.8)	0.129
24-hour urine volume (mL)	1,367.3 ± 1,026.2	1,689.3 ± 915.8	0.002
Intensive care requirement	16 (18.2)	94 (10.3)	0.032
Invasive MV used	7 (8)	18 (2)	0.004
Length of stay in intensive care (days)	1.1 ± 3.3	0.3 ± 1.6	0.000
Length of stay in the hospital (days)	13.2 ± 10.4	9.5 ± 6.9	0.000
Need for RRT (5 days)	1 (1.1)	0 (0)	0.088
Mortality (28 days)	2 (50)	3(13.6)	0.155

On univariate statistical analyses, the preoperative baseline characteristics of patients who developed and did not develop AKI after gynecological cancer surgery, as well as those that were statistically significant (p < 0.05) among the variables associated with intraoperative anesthesia and surgery, were brought together to determine the risk factors for postoperative AKI. These variables were included in the prospective logistic regression analysis first. After that, a reanalysis was performed with six parameters, including age, BMI, surgical procedure, intraoperative colloid used, ES, and FFP amounts which were thought to affect the clinical results. According to regression analysis, BMI and extensive surgery were found to be independent risk factors for AKI in the postoperative gynecological cancer surgery period. Extensive surgeries increased AKI progression risk by 2.3 times. Table 7 illustrates the odds ratios and the accompanying 95% confidence intervals which show the effect of these risk factors on AKI, in other words, the amount of contribution.

**Table 7:** Multivariate logistic regression analysis. Wald test statistics. Statistically significant p-values are in bold. OR = odds radio; CI = confidence interval; BMI = body mass index; ES = erythrocyte suspension; FFP = fresh frozen plasma

	OR	95% CI	P-value
Age (year)	1.012	0.993-1.032	0.218
BMI (kg/m^2^)	1.034	1.000-1.069	0.049
Extensive surgery	2.320	1.330-4.048	0.003
Total used colloid (mL/kg)	1.000	0.999-1.001	0.641
Total used ES (mL)	1.001	1.000-1.001	0.135
Total used FFP (mL)	1.000	0.999-1.001	0.994

## Discussion

Although there are many studies on postoperative AKI in cardiovascular surgery and kidney or liver transplantation, there is limited data on postoperative AKI incidence and risk factors for patients undergoing surgeries for gynecological cancer. This study aimed to retrospectively evaluate the kidney function of patients who underwent gynecological cancer surgery and to detect preoperative, intraoperative, and postoperative risk factors for AKI. In this study, postoperative AKI incidence was 8.8%. Moreover, BMI and extensive surgery were independent risk factors for AKI in patients who underwent gynecological cancer surgery.

According to previous studies, AKI incidence varies depending on the definition of AKI and the type of surgery performed. For example, even though AKI frequency was less than 1% in patients with normal preoperative kidney functions and who underwent non-cardiovascular surgery, AKI incidence was 8.5% in patients undergoing gastric bypass surgery for morbid obesity [5]. In the study of Brienza et al., postoperative AKI risk was 1% in non-cardiac surgery patients and 7% in ICU patients, while this rate was 32% in patients who underwent major surgery and were admitted to the ICU [6]. According to Ng et al., postoperative AKI incidence in cardiac surgery was 27% according to the Acute Kidney Injury Network classification [7]. In this study, the incidence of postoperative AKI was 8.8% according to the KDIGO 2012 criteria.

The impact of age on mortality is high because old patients have more comorbidities [8]. Moreover, Hilton et al. reported that the risk of AKI increases with rising age [9]. Another study that investigated the risk factors of AKI among patients in the ICU showed that old age increases the risk of AKI [10]. In addition, obesity was also the cause of many concomitant diseases. In a study examining 1,227 patients undergoing bariatric surgery, the risk of AKI increased with rising BMI [11]. Weingarten et al. reported that AKI developed in 167 (1.82%) of 9,171 patients who underwent arthroplasty, and high BMI was an independent risk factor [12]. In this study, preoperative demographic data of patients who underwent gynecological cancer surgery were investigated and were similar to other studies. The development of AKI was higher in older patients, those with a short height, and those with a high BMI. According to logistic regression analysis, high BMI was a risk factor; however, old age and short height were not considered to be risk factors.

Regarding CRP and AKI correlation, in a study evaluating patients who underwent percutaneous coronary intervention, it was found that preoperative high CRP is an independent risk factor for contrast-associated AKI [13]. Tang et al. investigated the impact of CRP on AKI and found that CRP has a pathogenic role in AKI by inhibiting tubular epithelial cell regeneration [14]. In this study, similarly, preoperative CRP values were higher in patients with AKI than those without AKI, but this was not identified as a risk factor. This can be attributed to the high incidence of ischemia/reperfusion injury in patients with high CRP levels.

Distant metastasis significantly affects the survival rates and prognosis. After surgeries, the probability of recurrence is also high in these patients. For patients with metastasis, the surgical approach is more invasive, and the surgical trauma risk is higher. One of the reasons for the rising AKI risk in patients with distant metastasis is extrarenal obstruction (retroperitoneal fibrosis, ureter or bladder outlet obstruction) [15]. Retroperitoneal tumor invasions might cause obstruction in the urinary tract. The most common cancers that cause this are cervix, bladder, colon, prostate, and ovarian cancers. Due to compression of the renal artery, hypoperfusion, ischemia, and hypertension might occur, and due to compression of the renal vein, nephrotic syndrome might develop [16]. In a study by Rosa et al., the incidence of AKI in 335 cancer patients was 27.7% according to the RIFLE criteria, with an incidence of 60.4% in patients with distant organ metastasis and 18.7% in those with limited organ involvement. AKI is particularly more common in patients with cervical, ovarian, prostate, breast, and gastric cancer [17]. Similarly, in this study, AKI was detected in 25 of 188 patients with distant organ metastasis. Statistical analysis showed a higher incidence of AKI in patients with distant organ metastasis preoperatively; however, this was not identified as an independent risk factor. This might be attributed to the wider tissue excision required in metastatic gynecological cancer surgeries.

Tachycardia is a common symptom in cancer patients and can be caused by anemia, hypovolemia, infection, medication use, or chemotherapy. In this study, the pulse rate was higher in patients with AKI compared to those without AKI when vital signs were examined preoperatively; however, this was not an independent risk factor for AKI. This condition might be attributed to tachycardia due to preoperative hypovolemia because one of the most common causes of AKI is hypoperfusion due to volume depletion. Early diagnosis and treatment of hypovolemia are known to be highly effective in preventing AKI [1].

As the surgeries become more invasive, the rate of complications and the duration of surgery increases. Multiple organs are affected in these procedures, making it difficult to maintain hemodynamic stability. In the study of Abelha et al., high-risk surgery was found to be an independent risk factor for AKI [18]. Brienza et al. also reported that intraperitoneal surgery increases the risk of AKI [6]. Although gynecological surgeries are not highly risky, AKI can occur after extensive surgical excision such as radical hysterectomy and debulking [1]. In the KDIGO 2012 guidelines, major surgery is listed as a risk factor for AKI [19]. In this study, the incidence of AKI was higher in those who underwent extensive surgery compared to those who did not. In addition, the surgical duration was found to be longer in patients who developed AKI compared to those who did not. The surgical duration was approximately half an hour longer (149.1 ± 62.5 vs. 123.8 ± 56.6 minutes) in postoperative AKI patients. However, while extensive surgery was found to be a risk factor in logistic regression analysis in our study, surgical duration was not identified as a risk factor.

It is known that colloids might have harmful effects on the kidneys [10]. In the study by Ishikawa et al., it was found that in 67 patients of 1,129 (5,6%) who underwent lung resection postoperative AKI developed and the use of intraoperative hydroxyethyl starch resulted in an increasing risk of AKI [20]. Fifty-nine hospitals contributed to a study examining 2,378 postoperative cases (incidence of AKI 1.1%) who underwent surgery, and blood loss and colloid infusion were found to be two out of the five risk factors for AKI (others are age, type of surgery, and presence of peripheral artery disease) [21]. Han et al. reported that intraoperative blood loss increases AKI risk because of hypotension [1]. The decrease in perfusion pressure caused by additional blood loss results in afferent arteriolar vasoconstriction, efferent arteriolar vasodilatation, and a decrease in glomerular hydrostatic pressure [22]. In addition, additional blood loss causes medullary hypoxia, which can lead to renal tubular necrosis and cause AKI [2]. In this study, comparing patients with and without AKI, we found that colloid, ES, and FFP use in patients with postoperative AKI was more than in those without postoperative AKI. Moreover, intraoperative blood loss was higher in patients with AKI. However, in this study, colloid, ES, and FFP use and blood loss were not considered risk factors according to regression analysis.

In a study investigating postoperative hypertension, it was found that hypertension usually begins within 30 minutes postoperatively and is mostly related to pain, hypercarbia, and anxiety [23]. In this study, when vital signs in the first 24 hours postoperatively were evaluated, hypertension and tachycardia were observed more frequently in patients with AKI. This situation is likely to have developed secondary to postoperative pain, but tachycardia might also have been caused by excessive fluid loss during the perioperative period.

Although colloids are better than crystalloids in maintaining intravascular volume, hyper-oncotic colloids have a higher risk of renal dysfunction compared to crystalloids and hypo-oncotic colloids [10]. In this study, although colloids were used more in the first postoperative 48 hours in patients with AKI, it was not considered a risk factor.

Complications might be observed in many systems in the postoperative period [24]. In a study examining 1,129 patients with lung resection, early postoperative AKI progression increased respiratory complication rate and length of hospital stay [20]. The most common postoperative hematological complications in patients with AKI were anemia, thrombocyte dysfunction, and bleeding. Chang et al. found that leukocytosis and thrombocytopenia were more common in patients with acute renal failure after endovascular thoracoabdominal aortic aneurysm repair compared to those without [25]. In this study, out of a total of 13 patients with postoperative hematologic complications detected with AKI, postoperative bleeding occurred in eight patients, anemia in seven patients, and disseminated intravascular coagulation in one patient. Postoperative respiratory complications developed in 31 patients, and AKI was detected in seven of these patients (two respiratory failures, three pleural effusions, one hypoxia-atelectasis, and one pneumonia). Fluid therapy administered to those who developed AKI might lead to the development of respiratory complications, and hypoxia resulting from respiratory complications might also increase the risk of AKI in these patients.

AKI has a significant impact on the length of stay in the ICU [18]. Cabezuelo et al. studied risk factors for acute kidney failure after liver transplantation in 184 cadavers and found that mechanical ventilation requirements and prolonged stay in the ICU were postoperative variables that increased the risk of acute kidney failure [26]. In a study among children undergoing surgery due to congenital heart disease, mechanical ventilation was found to be an independent risk factor for mortality in patients with AKI, and these patients required mechanical ventilation for a longer duration compared to those without AKI [27]. In this study, an increase in the need for ICU, mechanical ventilation, and length of stay in the ICU was observed in patients with AKI compared to those without AKI; however, these were not identified as independent risk factors. The need for mechanical ventilation and ICU in patients with AKI might be attributed to respiratory complications or higher amounts of total colloid, ES, and TDP used during surgery. There was a need for ICU due to kidney failure.

In a study by Chertow et al., length of hospital stay was reported as an independent risk factor for AKI [28]. In another study by Waikar et al., the average length of hospital stay for patients with AKI was seven days, and there was an average increase of two days in the length of stay for those who developed AKI [29]. A study conducted to investigate the impact of AKI on long-term mortality retrospectively examined patients at least 90 days after discharge and found a higher incidence of AKI in patients who stayed in the hospital for a longer duration [30]. In this study, the length of hospital stay was longer in patients who developed AKI compared to those who did not, but it was not identified as a risk factor for AKI. The mean length of hospital stay for patients with AKI was 13.2 ± 10.4 days, while among those without AKI, it was 9.5 ± 6.9 days.

Considering that patient follow-up and records might vary between different centers, differences in risk factors for AKI obtained from studies can be explained. Additionally, differences in scoring systems used to diagnose AKI in studies can also explain this variability. According to the logistic regression analysis results, in this study, BMI and extensive surgery were independent risk factors for AKI after gynecological cancer surgery. The application of extensive surgery increased the risk of AKI by 2.3 times among these risk factors.

When considering our study methodologically, it contains limitations of retrospective studies as certain patient information, such as preoperative and postoperative laboratory data, the need for renal replacement therapy in some patients, and intraoperative urine output, were not accessible during screening. The data were screened based on the reliability of the medical records and patients with incomplete information that could significantly affect the results were excluded from the evaluation to ensure no data gaps in the statistical analysis.

## Conclusions

It is important to determine preoperative, intraoperative, and postoperative risk factors for AKI, which is an important problem affecting postoperative mortality and morbidity, to take necessary precautions, as well as to detect and intervene early in AKI. Advanced age, high BMI, high preoperative CRP levels, distant organ metastasis, extensive surgery, long operation times, and high intraoperative blood transfusion were found to be independent risk factors for postoperative AKI in patients undergoing gynecological cancer surgery. We believe that the incidence of AKI can be reduced by conducting comprehensive prospective studies including more patients and different age groups of patients undergoing cancer surgery, determining the risk factors for postoperative AKI, and taking precautions.
